# Roles of Interleukin‐24 in Epithelial Repair: Bridging Injury and Regeneration

**DOI:** 10.1111/all.16479

**Published:** 2025-01-21

**Authors:** Morgan Bryant, Piotr P. Janas, Thibaut Sanchez

**Affiliations:** ^1^ School of Medicine, Medical Sciences and Dentistry, Institute of Medical Sciences University of Aberdeen Aberdeen UK; ^2^ Centre for Inflammation Research, Institute for Regeneration and Repair The University of Edinburgh Edinburgh UK

1

The first line of defence against invading pathogens consists of the barrier epithelial tissues of the skin, gut and lungs. Upon injury, blood clot formation takes place, followed by the infiltration of immune cells that remove debris and subsequently promote tissue repair mechanisms. Structural cells then proliferate to rebuild lost tissue, and finally, the wound undergoes remodelling to restore the tissue architecture and function [1].

It has been long understood that disruption of skin integrity is often accompanied by pathogen invasion, resulting in an inflammatory response, which triggers the initial immune response commencing wound healing [2]. These initial wound repair stages are followed by tightly coordinated migration and proliferation of epidermal progenitors to restore tissue integrity. However, until recently, the exact signalling pathways allowing for coordination of re‐epithelialisation, especially in the absence of pathogens, remained elusive.

The study conducted by Liu et al. [3] aimed to investigate the repair mechanisms following skin injury that are independent of the response to pathogen infection. Using a mouse model of skin wound healing, the group identified epithelial‐specific increased phosphorylation of the transcription factor STAT3, accompanied by upregulation of IL‐24 (Figure 1). Increased IL‐24 appeared independent of microbial responses, as wounding in either germ‐free mice or mice lacking TLR signalling still led to IL‐24 upregulation. The authors hypothesised that IL‐24 induction is mediated by epidermal stem cells (EpdSC) sensing ‘non‐homeostatic’ patterns that are independent of pathogen‐induced interferon signalling pathways.

**FIGURE 1:**
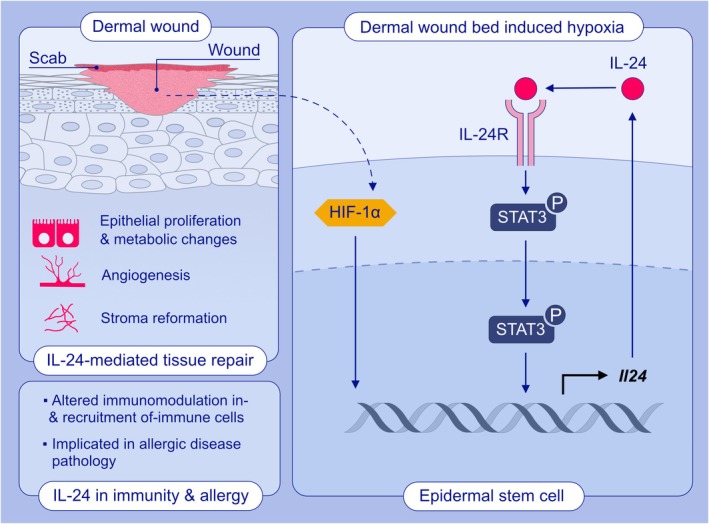
Epidermal‐derived IL‐24 orchestrates a coordinated repair response in a model of murine skin wound healing. Wound‐induced hypoxia triggers HIF‐1α–dependent *Il24* expression in epidermal stem cells. IL‐24 acts via autocrine and paracrine signalling further promoting self *Il24* expression through an IL‐24R–STAT3–*Il24* signalling axis. IL‐24 signalling induces epithelial proliferation, metabolic changes, stromal reformation and revascularisation of the wound bed. Additionally, IL‐24 modulates immune cells present in the wound environment, influencing their recruitment and impacting repair outcomes. Interestingly, IL‐24 has also been implicated in the pathogenesis of allergic dermatitis, highlighting its broader role in skin‐related inflammatory processes.

Following the deletion of *Il24* or its receptor subunit, *Il20rb*, phosphorylation of STAT3 was markedly reduced around the wound edge, highlighting that IL‐24 acts upstream of STAT3 in response to tissue damage. A deficiency in IL‐24 signalling not only reduced epithelial proliferation but also distinctly impaired revascularisation and fibroblast‐mediated stroma regeneration, leading to delayed wound closure. This effect was linked to the injury‐induced hypoxic epithelial niche, which led to increased HIF1α expression in EpdSCs, further promoting IL‐24 expression. In vitro studies further confirmed that a substantial increase in IL‐24 expression was seen in hypoxic conditions.

Finally, the authors demonstrated that the HIF‐1α‐IL‐24‐STAT3 signalling axis causes metabolic changes in cells at the wound edge. The expression of a specific glucose transporter, GLUT1, appeared to be sensitive to the expression of IL‐24, its receptor and subsequent signalling cascade. This transporter was found to play an important role in restoring the endothelial and fibroblast cell niche near the wound edge. Therefore, in response to hypoxic conditions arising following tissue injury, EpdSCs express IL‐24 that alters their metabolism, migration and proliferation while orchestrating a multi‐cellular response.

Different cell types present in the wound, other than EpdSCs, are known to be directly or indirectly sensitive to IL‐24 [4]. These include immune cells such as macrophages, in which IL‐24 induces an anti‐inflammatory response [5]. T lymphocytes are also sensitive to this cytokine, but the associated cellular responses may have varying effects, which can in turn influence the recruitment of other immune cells such as neutrophils or monocytes affecting tissue repair outcomes [4].

IL‐24 plays a significant role in several allergic diseases. In a model of allergic contact dermatitis (ACD), IL‐24 expression is upregulated, further implicating it in skin immune regulation [6]. Mice with deficient IL‐24 signalling have impaired neutrophil recruitment and are protected from developing chemical‐induced contact hypersensitivity. IL‐24 acting detrimentally in ACD and other pathologies emphasises the need to better understand its immunomodulatory role.

Developing novel therapies for chronic wounds and scarring requires advancing our understanding of IL‐24's role. This includes gaining a deeper insight into its effects on various cell types within the wound niche and exploring how IL‐24 signalling intersects with other tissue repair mechanisms, particularly in the context of infection or allergy.

## Conflicts of Interest

The authors declare no conflicts of interest.

## Data Availability

The authors have nothing to report.
